# Genome-wide DNA methylation analysis of pituitaries during the initiation of puberty in gilts

**DOI:** 10.1371/journal.pone.0212630

**Published:** 2019-03-07

**Authors:** Xiaolong Yuan, Zhonghui Li, Shaopan Ye, Zitao Chen, Shuwen Huang, Yuyi Zhong, Hao Zhang, Jiaqi Li, Zhe Zhang

**Affiliations:** National Engineering Research Center for Breeding Swine Industry, Guangdong Provincial Key Lab of Agro-Animal Genomics and Molecular Breeding, College of Animal Science, South China Agricultural University, Guangzhou, Guangdong, China; UNC Eshelman School of Pharmacy, UNITED STATES

## Abstract

It has been widely recognized that the early or delayed puberty appears to display harmful effects on adult health outcomes. During the timing of puberty, pituitaries responds to the hypothalamus and then introduce the following response of ovaries in hypothalamic-pituitary-gonadal axis. DNA methylation has been recently suggested to regulate the onset of puberty in female mammals. However, to date, the changes of DNA methylation in pituitaries have not been investigated during pubertal transition. In this study, using gilts as the pubertal model, the genome-scale DNA methylation of pituitaries was profiled and compared across Pre-, In- and Post-puberty by using the reduced representation bisulfite sequencing. We found that average methylation levels of each genomic feature in Post- were lower than Pre- and In-pubertal stage in CpG context, but they were higher in In- than that in Pre- and Post-pubertal stage in CpH (where H = A, T, or C) context. The methylation patterns of CpHs were more dynamic than that of CpGs at the location of high CpG content, low CpG content promoter genes, and differently genomic CGIs. Furthermore, the differently genomic CGIs were likely to show in a similar manner in CpG context but display in a stage-specific manner in the CpH context across the Pre-, In- and Post-pubertal stage. Among these pubertal stages, 5 kb upstream regions of the transcription start sites were protected from both CpG and CpH methylation changes. 12.65% of detected CpGs were identified as the differentially methylated CpGs, regarding 4301 genes which were involved in the fundamental functions of pituitaries. 0.35% of detected CpHs were identified as differentially methylated CpHs, regarding 3691 genes which were involved in the biological functions of releasing gonadotropin hormones. These observations and analyses would provide valuable insights into epigenetic mechanism of the initiation of puberty in pituitary level.

## Introduction

In female mammals, the initiation of puberty indicates the achievements of adult height, body proportion, and the capacity of reproduction [1, 2]. In women, it has been widely recognized that the early or delayed puberty appears to display harmful effects on adult health outcomes [3–5], but the underlying molecular mechanism of pubertal timing has been largely unknown in mammals. Generally, the timing of puberty is driven by the hypothalamic-pituitary-gonadal (HPG) axis in female mammals [6, 7]. An increase in the pulsatile release of gonadotropin-releasing hormone from the hypothalamus results in increased luteinizing hormone and follicle-stimulating hormone release from the pituitary [6, 7], and both luteinizing hormone and follicle-stimulating hormone act on the folliculogenesis, oogenesis, and sex steroid production of the gonads [8–10]. These observations indicate that the pituitary exhibits a pivotal role during the onset of puberty, which responds to hypothalamus and then introduces the following response of ovaries in HPGs axis.

DNA methylation is one of the most investigated epigenetics. Importantly, high CpG content promoters (HCPs) and low CpG content promoters (LCPs), showing essentially differentially methylated and expression patterns [11, 12], are strongly associated with housekeeping genes and tissue-specific genes [13, 14], respectively. Additionally, CpG islands (CGIs) are frequently identified as the potential promoters [15] and the regulatory element [16] to support the transcription of genes, and the methylation of CGIs is closely interacted with the methylation of genes [17]. Currently, DNA methylation has been strongly suggested to control and regulate the onset of puberty in female mammals [18, 19]. Previous studies have reported that changes of DNA methylation cause *KISS1* gene, which has emerged as an essential gatekeeper for the onset of puberty via directing the stimulation of GnRH secretion at the hypothalamic level [20–22], switching from repressive to permissive in mice [19, 23, 24]. Moreover, the changes and dynamics of genome-wide DNA methylation during the onset of puberty have been described for the hypothalamus of female goats [25, 26] and rats [27], and these studies have provided useful insights into the epigenetic mechanism for the timing of puberty at hypothalamus level for mammals. However, few investigations have focused on the dynamics and changes of DNA methylation in pituitaries during the pubertal transition in mammals.

In pigs, puberty is defined as the emergence of the first estrous [28], and furthermore, the gilts are the valuable biomedical and pubertal model due to the similar physiological characteristics during the initiation of puberty with humans [29, 30]. In this study, to investigate the changes of DNA methylation in pituitaries during the pubertal transition, using pigs as the pubertal model, the genome-scale DNA methylation of pituitary was generated and profiled for Pre-, In- and Post-puberty by using the reduced representation bisulfite sequencing (RRBS). First, these methylation profiles were compared to describe changes for gene- and CGI-related regions in both CpG and CpH (where H = A, T, or C) contexts. Then the dynamic DNA methylation were investigated and profiled for the whole genome, HCP genes, LCP genes, and differently genomic CGIs across Pre-, In- and Post-puberty, respectively. Subsequently, these methylation dynamics were identified to explore their biological functions. This work will provide valuable insights into the epigenetic mechanism for the timing of mammalian puberty at pituitary level.

## Materials and methods

### Ethics statement

The pig cares and experiments were approved by the Animal care and Use Committer of South China Agricultural University, Guangzhou, China (approval number: SCAU#2013–10), and conductions were based on the Regulations for the Administration of Affairs Conerning Experimental Animals (Ministry of Science and Technology, China, revised in Jun 2004). The Pigs were fed the same diet daily and reared in the same conditions and environments, and pigs were pre-treated with anesthetic induction to alleviate suffering. The pituitaries of each pubertal group were collected, frozen quickly in liquid nitrogen and then stored at -80°C for further using.

### Animals and sample preparation

The pituitary samples were collected from Landrace × Yorkshire crossbred gilts. In gilts, the onset of puberty could be handily identified by the standing reflex with the back-pressure test and boar contact [31]. 25 Landrace × Yorkshire crossbred gilts aged at 160 days were selected and used in this study. Pubertal signs were checked and recorded twice daily at 09:00 and 15:30 by inspection of the vulva and assessment of the standing reflex for these 25 gilts. Three gilts aged at 180 days without pubertal signs were selected as Pre-pubertal gilts; three gilts at the day exhibiting the first estrous and the standing reflex were selected as the In-pubertal gilts (about 205 days); and another three gilts in the dioestrus phase, 14 days after the day exhibiting the first estrus and standing reflex, were selected as the Post-pubertal gilts.

### Constructions of RRBS libraries

The library constructions and sequencing services were provided by RiboBio Co., Ltd. (Guangzhou, China), which were previously described in our studies [32, 33]. The genomic DNA of these pituitary tissues was extracted using a DNeasy Blood & Tissue Kit (Qiagen, Beijing), and then, after checking on the quality of the extracted DNA, one library was built for each gilt based on previously published RRBS studies [17, 32, 33]. The processes and procedures of RRBS libraries were briefed as follows. Firstly, the purified genomic DNA was digested overnight with *Msp*I (New England Biolabs, USA). For the *Msp*I digested segments, the sticky ends were filled with CG nucleotides and 3′ A overhangs were added. Secondly, methylated Illumina sequencing adapters with 3′ T overhangs were ligated to the digested segments, and the products obtained were purified. Then 110–220 bp fragments were selected [32] and converted by bisulfite using an EZ DNA Methylation Gold Kit (Zymo Research, USA). Lastly, libraries of 110–220 bp fragments were PCR amplified and each library was sequenced using one lane of an Illumina HiSeq 2500 and 100 bp paired-end reads.

### Bioinformatic and data analysis of RRBS

The first two nucleotides were trimmed from all the second read sequences to blunt-end the MspI site. All reads were trimmed using Trim Galore (v0.4.0) software (Babraham Bioinformatics, http://www.bioinformatics.babraham.ac.uk/projects/trim_galore/) and a Phred quality score of 20 as the minimum. The adaptor pollution reads and multiple N reads (where N >10% of one read) were removed to generate the clean reads. The quality control checks were performed by FastQC (v0.11.3) software (Babraham Bioinformatics). The clean reads were mapped to the pig reference genome (Sscrofa 11.1, downloaded from Ensembl, http://asia.ensembl.org/Sus_scrofa/Info/Index), and then, the DNA methylation calling was performed by Bismark (v0.14.5) [34] using the default parameters.

263.94 million paired-end clean reads were totally generated for these nine libraries, and 29.33 million paired-end clean reads were generated for each library. These nine RRBS data were submitted to European Nucleotide Archive. After DNA methylation calling by Bismark [34] for these nine RRBS datasets, CpGs or CpHs covered by at least five reads and co-detected across all tissues were remained for further analysis. The methylation level of the CpGs or CpHs was calculated as the methylated reads divided the total covered reads. The methylation level of one group of pituitaries was calculated by the average methylation level across the three replicates.

For each specific region, the methylation level was measured as the average level of CpGs or CpHs located in this region. To profile the DNA methylation patterns at the gene and CGI locations, the genic locations were divided into 20, 40 and 20 bins for 5 kb upstream region of the transcription start sites (TSSs), gene body and 5 kb downstream region of transcription end sites (TESs), respectively, and the CGI locations were divided into 20, 20 and 20 bins for 2 kb upstream region, CGIs and 2 kb downstream region, respectively. These analyses were performed by Perl and R scripts. The bisulfite conversion rates were calculated as the number of covered CpHs, which were unconverted, was divided by the total number of covered CpHs [35]. The bisulfite conversion efficiencies of these nine libraries were 99.47%, 99.57%, 99.42%, 99.39%, 99.26%, 99.45%, 99.31%, 99.34%, and 99.43% for Pre-puberty 1, Pre-puberty 2, Pre-puberty 3, In-puberty 1, In-puberty 2, In-puberty 3, Post-puberty 1, Post-puberty 2, and Post-puberty 3, respectively. This pipeline was carefully described by our previous studies [17, 33].

### Annotation of genes and CGIs

The genic locations were downloaded from Ensembl (http://asia.ensembl.org/Sus_scrofa/Info/Index). Basing on genic locations of Ensembl, the porcine genome was separated into five genic features, which were upstream, exonic, intronic, downstream and intergenic regions. The upstream region was 5 kb upstream region of the TSS. The exon was defined as the integration of 5′UTR, CDS and 3′UTR arranging from the TSS to the TES. The intron was determined as the integration of introns arranged from the TSS to the TES. The downstream region was 5 kb downstream region of the TES. The intergenic region was denoted as the outside regions of upstream, exonic, intronic and downstream regions.

The locations of CGIs in pigs were downloaded from UCSC (http://hgdownload.soe.ucsc.edu/goldenPath/susScr11/database/). CGIs were described as regions >200 bp in length, with a C and G percentage >0.5, and a ratio of the observed CpG/expected CpG >0.6. The expected CpG was calculated as the number of Cs multiplied by the number of Gs, divided by the length of the segment. The +/− 2 kb regions outside of CGIs were defined as CGI shores, and the +/− 2 kb regions outside of CGI shores were defined as CGI shelves.

We previously localized CGIs to genic features [17], based on the localization of CGIs and genic features. Briefly, when more than 50% of a CGI overlapped with a specific genic feature, that CGI was classified with the specific genic feature. For example, when the overlap ratio between the location of a CGI and the downstream region was greater than 50%, that CGI was defined as a downstream CGI. According to this process, the CGI was annotated as Upstream-CGI, Intronic-CGI, Exonic-CGI, Downstream-CGI and Intergenic-CGI. The processes and procedures of CGI annotation were detailly described in our previous study [17].

### Statistics analysis

According to our previous study, the porcine genes could be separated into two classes: HCP and LCP genes [33]. The significant differences among these three stages were tested using the variance analysis with the function of “anova”, the enrichment was tested using a two-tailed Fisher’s exact test with the function of “fisher.test”, and the Pearson’s correlation coefficient was tested with the function of “cor.test” in R “stats” package (https://www.rdocumentation.org/packages/stats/).

The differentially methylated CpG sites (DMGs) and CpH sites (DMHs) were calculated by CGmapTools [36]. The CpGs or CpHs whose methylation levels changed more than 20% were identified as DMGs or DMHs according to a two-tail Fisher’s exact test (*P* ≤ 0.05) [36]. The genes, including 5 kb upstream flanking region, gene body and 5 kb downstream flanking region, overlapped at least one DMG or DMH were defined as the DMG or DMH regarding genes, respectively. For the comparison between two groups, DMGs or DMHs with higher methylation level in one group were considered as the hyper-methylated DMGs or DMHs in this group and were considered as the hypo-methylated DMGs or DMH in another group, respectively. The enrichment for certain genomic regions was calculated by using a two-tail Fisher’s exact test. The Gene Ontology (GO) and Kyoto Encyclopedia of Gene and Genomes (KEGG) enrichment analysis were undertaken by DAVID (https://david.ncifcrf.gov/home.jsp) [37].

## Results

### Genome-wide DNA methylation of pituitary tissues during the timing of puberty

**Fig 1:**
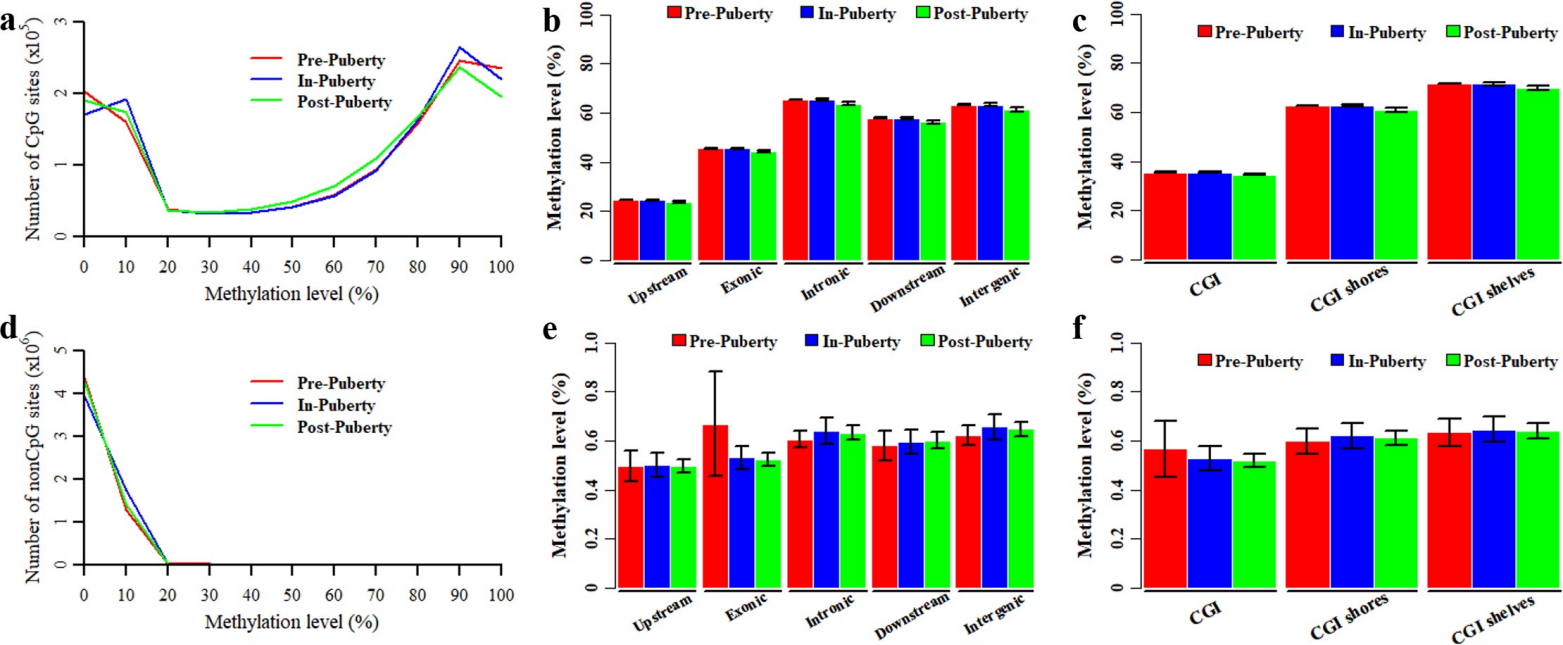
Genome-wide DNA methylation of pituitary tissues during the timing of puberty. Distribution of methylation levels in CpG (**a**) and CpH (**d**) context. Average methylation levels of CpGs in gene- (**b**) and CGI-related regions (**c**). Average methylation levels of CpHs in gene- (**e**) and CGI-related regions (**f**).

In the CpG context, 1301936 CpGs covered with at least five reads detected in all nine libraries were selected for further analysis. The average methylation levels were 53.51% ± 0.15%, 53.42% ± 0.40% and 52.04% ± 0.62% for Pre-, In- and Post-puberty, respectively. The CpG methylation levels all presented a bimodal distribution (Fig 1A), but the distributed features of these three stages could be clearly distinguished from each other in the bimodal peaks (Fig 1A). Comparisons of these stages revealed that Post-puberty displayed the most of CpGs with methylation level ≤ 10% (Pre: 28.21% ± 0.21%; In: 28.28% ± 0.08%; Post: 28.50% ± 0.25%) and the most of CpGs with methylation level ranging from 50% to 80% (Pre: 24.33% ± 0.10; In: 24.27% ± 0.67%; Post: 26.01% ± 0.34%) but displayed the lowest of CpGs with methylation level≥90% (Pre: 20.43% ± 0.94%; In: 18.89% ± 0.94%; Post: 17.89% ± 0.56%). The average methylation levels of detected CpGs within the different genomic features were shown in Fig 1B and 1C. We found that the average methylation levels of each genomic feature in Post- were lower than that in Pre- and In-puberty, although these differences were insignificant (Fig 1B and 1C). Besides, the average methylation levels of CGIs and upstream regions were observed to be lower than other CGI- and gene-related regions, respectively (Fig 1B and 1C).

In the CpH context, 5708233 CpHs covered with at least five reads detected in all nine libraries were selected for further analysis. The average methylation levels of CpH methylome were 0.60% ± 0.07%, 0.61% ± 0.05% and 0.60% ± 0.03% for Pre-, In- and Post-puberty, respectively. The numbers of detected CpHs with methylation level ≤20% were 99.70% ± 5.49%, 99.81% ± 0.01% and 99.75% ± 0.05% for Pre-, In- and Post-pubertal stages, respectively (Fig 1D). Comparisons of these stages revealed that the average methylation levels of Upstream, Intronic, Downstream, Intergenic, CGI shores and CGI shelves regions were the highest in In-pubertal methylome but were the lowest in Pre-pubertal (Fig 1E and 1F). However, the average methylation levels of CGI and Exonic region were the highest in Pre- and the lowest in Post-pubertal stage (Fig 1E and 1F).

### Global DNA methylation dynamics among these pituitaries

**Fig 2:**
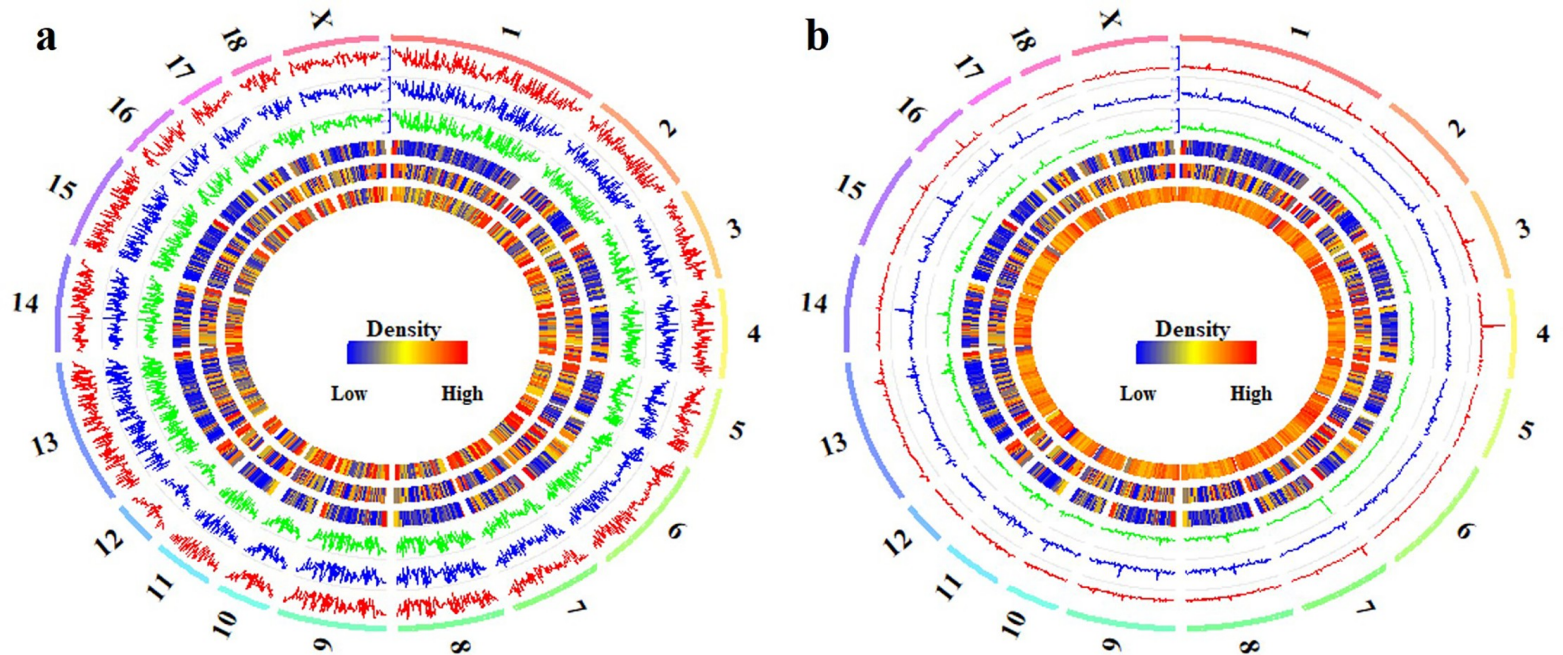
Global methylation of CpGs and CpHs in the pituitaries. The global CpG (a) and CpH (b) methylation levels in Pre- (track 1), In- (track 2) and Post-pubertal stage (track 3) of the pituitary tissue, from outside to inside, were quantified per 1Mb window. The densities of CGIs (track 4), genes (track 5) and CpGs (track 6 in **a**) or CpHs (track 6 in **b**) were also quantified per 1 Mb window. The labels outside of track 1 represent the chromosomes of the porcine genome.

To explore the methylation dynamics during the onset of puberty, the DNA methylation levels and the densities of CpGs and CpHs in the pituitaries were profiled per 1 Mb in Fig 2. In the CpG context, the Pearson’s correlation coefficients of methylation levels were all 0.99 (*P* < 2.22 × 10^−16^) for Pre- vs. In-, In- vs. Post-, and Pre- vs. Post-pubertal methylome (Fig 2A and S1 Table). The CpG methylation of Pre-, In-, and Post-pubertal pituitary were all positively correlated with the densities of CpGs (Pearson’s correlation coefficient = 0.22, *P* < 2.22 × 10^−16^) and CGIs (Pearson’s correlation coefficient = 0.27, *P* < 2.22 × 10^−16^) (Fig 2A and S1 Table), but negatively correlated with the densities of genes (Pearson’s correlation coefficient = -0.12, *P* < 2.22 × 10^−16^) (Fig 2A and S1 Table). In the CpH context, however, the Pearson’s correlation coefficient was 0.57, 0.80 and 0.58 for Pre- vs. In-, In- vs. Post-, and Pre- vs. Post-stage in CpH context, respectively (Fig 2B and S1 Table). The Pearson’s correlation coefficient was 0.10 (*P* < 2.22 × 10^−16^), 0.15 (*P* < 2.22 × 10^−16^) and 0.15 (*P* < 2.22 × 10^−16^) between the densities of CpHs and the CpH methylation of Pre-, In- and Post-puberty (Fig 2B and S1 Table), and the Pearson’s correlation coefficient was 0.07 (*P* < 2.22 × 10^−16^), 0.15 (*P* < 2.22 × 10^−16^) and 0.14 (*P* < 2.22 × 10^−16^) between the densities of CGIs and the CpH methylation of Pre-, In- and Post- puberty, respectively (Fig 2B and S1 Table). The Pearson’s correlation coefficient was -0.01 (*P* < 2.22 × 10^−16^), -0.07(*P* < 2.22 × 10^−16^) and -0.06 (*P* < 2.22 × 10^−16^) between the densities of genes and the CpH methylation in Pre-, In- and Post- puberty, respectively (Fig 2B and S1 Table).

### Methylation dynamics in the location of genes and CGIs among these pituitaries

**Fig 3:**
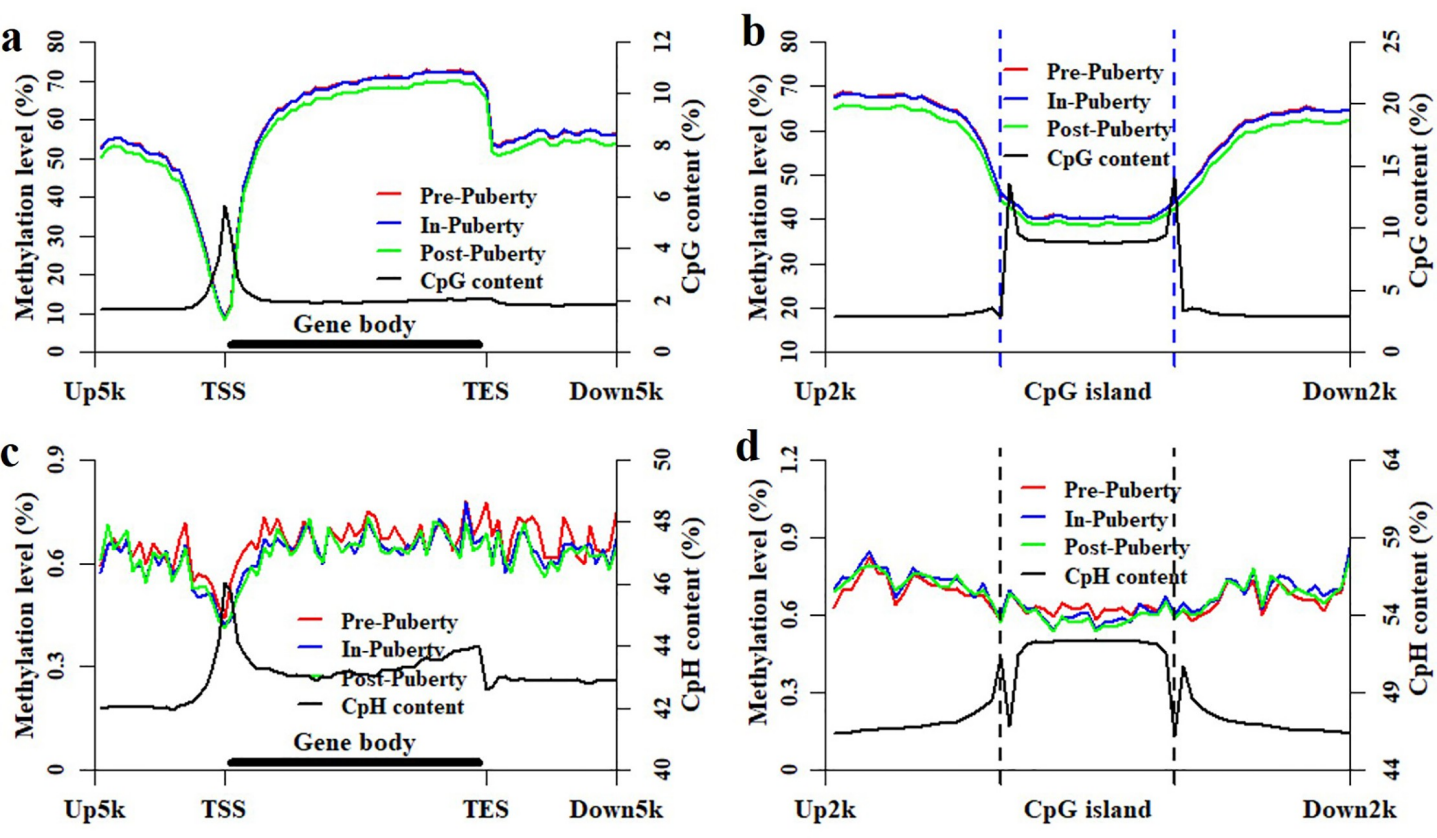
Methylation and density patterns of CpGs and CpHs at the locations of genes and CGIs. Methylation and density patterns of CpGs at the locations of genes (**a**) and CGIs (**b**). Methylation and density patterns of CpHs at the locations of genes (**c**) and CGIs (**d**).

To investigate the methylation dynamics at the locations of genes and CGIs in these pituitaries, the methylation patterns of CpGs and CpHs were profile as well as the densities of CpGs and CpHs (Fig 3). Compared with that in Pre- and In-stage, the CpG methylation level was the lowest at the locations of genes and CGIs in Post-stage (Fig 3A and 3B), and the methylation level of Pre- was highly overlapped with that of In-stage (Fig 3A and 3B). The methylated patterns were all highly correlated with each other at the locations of genes (Person’s correlation coefficient = 0.99, *P* < 2.22 × 10^−16^) and CGIs (Person’s correlation coefficient = 0.99, *P* < 2.22 × 10^−16^) for the comparison of Pre- vs. In-, Pre- vs. Post- and In- vs. Post-puberty, respectively (Fig 3A and 3B). The methylation patterns of Pre-, In-, and Post-puberty were all highly negatively correlated with the densities of CpGs at the locations of genes (Person’s correlation coefficient = -0.61, *P* ≤ 1.51 × 10^−9^) and CGIs (Person’s correlation coefficient = -0.85, *P* < 2.22 × 10^−16^), respectively (Fig 3A and 3B).

In the CpH context, the methylation levels were likely to be overlapped together across Pre-, In- and Post-puberty at gene and CGI locations (Fig 3C and 3D). At the genic locations, the Person’s correlation coefficient was 0.88 (*P* < 2.22 × 10^−16^), 0.81 (*P* < 2.22 × 10^−16^) and 0.95 (*P* < 2.22 × 10^−16^) for Pre- vs. In-, Pre- vs. Post- and In- vs. Post-stage, respectively (Fig 3C and 3D and S2 Table). At the CGI locations, the Person’s correlation coefficient was 0.92 (*P* < 2.22 × 10^−16^), 0.89 (*P* < 2.22 × 10^−16^) and 0.97 (*P* < 2.22 × 10^−16^) for Pre- vs. In-, Pre- vs. Post- and In- vs. Post-stage, respectively (Fig 3C and 3D and S2 Table). Additionally, the Person’s correlation coefficient was -0.17 (*P* = 0.14), -0.20 (*P* = 0.07), and -0.28 (*P* = 0.01) between the methylation patterns of CpHs and the densities of CpHs at genic location for Pre-, In-, and Post-puberty, respectively (Fig 3C and S2 Table). The Person’s correlation coefficient was -0.59 (*P* = 7.56 × 10^−7^), -0.78 (*P* = 2.16 × 10^−13^), and -0.80 (*P* = 7.54 × 10^−15^) between the methylation patterns of CpHs and the densities of CpHs at CGI locations for Pre-, In-, and Post-puberty, respectively (Fig 3D and S2 Table).

### Methylation patterns of HCP and LCP genes among these pituitaries

**Fig 4:**
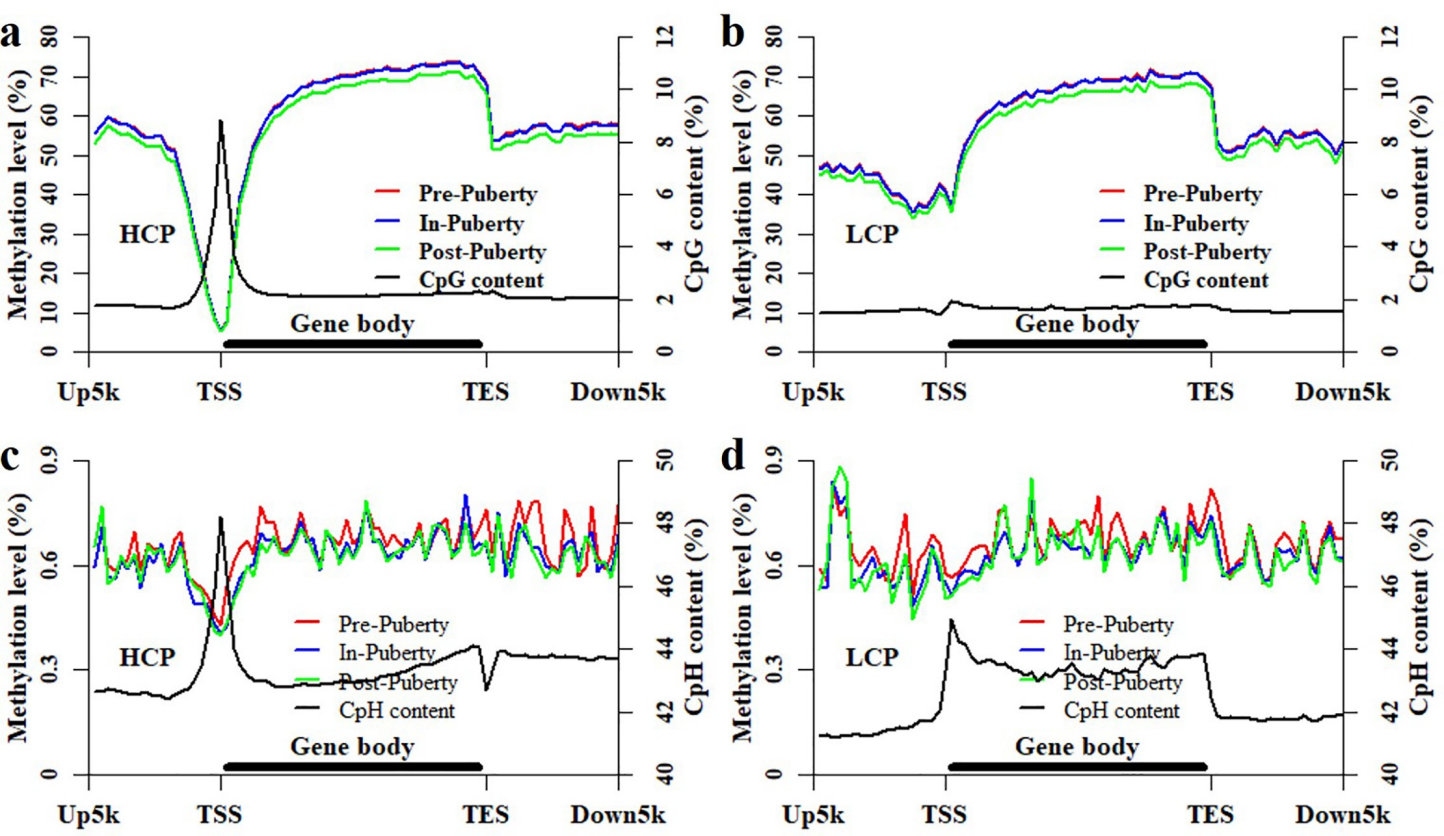
Methylation and density patterns of CpGs and CpHs at the locations of HCP and LCP genes. Methylation and density patterns of CpGs at the locations of HCP (**a**) and LCP (**b**) genes. Methylation and density patterns of CpGs at the locations of HCP (**c**) and LCP (**d**) genes.

Many previous studies reported that the methylated pattern of HCP genes was differently from that of LCP genes [12, 33]. According to our previous study, the porcine genes could be separated into two classes: HCP and LCP genes [33]. To further explore the dynamics at the locations of genes, the methylation patterns of CpGs and CpHs were profile as well as the densities of CpGs and CpHs for HCP and LCP genes (Fig 4), respectively. We found that the methylation levels of HCP and LCP genes displayed the same patterns to that in Fig 3A and 3B with the observations that the CpG methylation level was the lowest in Post-puberty (Fig 4A and 4B), and the Pre- highly overlapped with the In-puberty. For the comparisons of Pre- vs. In-, Pre- vs. Post- and In- vs. Post-stage, the methylation patterns of CpGs were highly correlated with each other at the locations of HCP (Person’s correlation coefficient = 0.99, *P* < 2.22 × 10^−16^) and LCP (Person’s correlation coefficient = 0.99, *P* < 2.22 × 10^−16^) genes, respectively (Fig 4A and 4B). Besides, the methylation patterns of CpGs in Pre-, In- and Post-puberty were all negatively correlated with its CpG density (Person’s correlation coefficient = -0.69, *P* < 0.74 × 10^−12^) at the locations of HCP genes (Fig 4A) but were all positively correlated with its CpG density (Person’s correlation coefficient = 0.46, *P* < 1.22 × 10^−5^) at the locations of LCP genes (Fig 4B).

However, the Person’s correlation coefficients between the CpH methylation patterns were 0.85, 0.93 and 0.80 at the locations of HCP genes (Fig 4C and S3 Table), and 0.86, 0.94 and 0.79 at the locations of LCP genes (Fig 4D and S3 Table) for the comparison of Pre- vs. In-, In- vs. Post-, and Pre- vs. Post-pubertal stage, respectively. The Person’s correlation coefficient between the CpH methylation pattern and density of CpHs was -0.36, -0.43 and -0.51 at the locations of HCP genes (Fig 4C and S3 Table), and it was 0.13, 0.15 and 0.13 at the locations of LCP genes (Fig 4D and S3 Table) for the Pre-, In- and Post-puberty, respectively.

### Methylation patterns of differently genomic CGIs among these pituitaries

**Fig 5:**
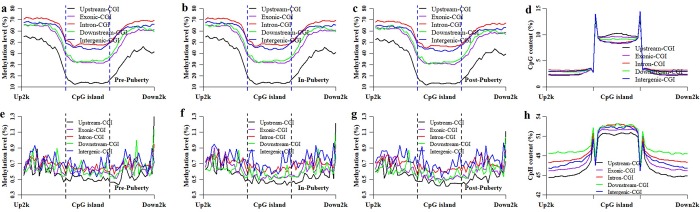
Methylation patterns of differently genomic CGIs. The CpG methylation patterns of Upstream-, Exonic-, Intronic-, Downstream-, and Intergenic-CGIs in Pre- (**a**), In- (**d**) and Post- (**c**). The CpG (**d**) and CpH (**h**) densities patterns of Upstream-, Exonic-, Intronic-, Downstream-, and Intergenic-CGIs in the porcine genome. The CpH methylation patterns of Upstream-, Exonic-, Intronic-, Downstream-, and Intergenic-CGIs in Pre- (**e**), In- (**f**) and Post-pubertal stage (**g**).

We previous found that the differently genomic CGIs exhibited different methylation patterns in pigs [17]. To further investigate the methylation dynamics of CGIs, the methylation patterns of differently genomic CGIs were profiled for these pituitaries in Fig 5. In the CpG context, the methylation levels of Intronic-CGIs were the highest (Fig 5A, 5B, and 5C), and Upstream-CGIs were the lowest (Fig 5A, 5B and 5C). The methylation levels of Exonic- and downstream-CGIs were lower than that of Intergenic-CGIs (Fig 5A, 5B and 5C). Moreover, the CpG methylation of these different CGIs trended to be reversed correlation with its densities of CpGs (Fig 5D), and the methylation patterns among Upstream-, Exonic-, Intronic-, Downstream- and Intergenic-CGIs were likely to show in a similar manner for the Pre-, In- and Post-pubertal stage (Fig 5A, 5B and 5C).

However, in the CpH context, the methylation levels and patterns among Upstream-, Exonic-, Intronic-, Downstream- and Intergenic-CGIs could not be distinguished from each other in Pre-puberty. In In- and Post-pubertal stage, the CpH methylation levels of Intergenic- and Intronic CGIs were likely to be higher than that of Exonic and Downstream-CGIs, and Upstream-CGIs presented the lowest the methylation levels (Fig 5F and 5G). The CpH methylation levels of these different CGIs trended to be reversed correlation with its densities of CpHs, with exception of Downstream-CGIs (Fig 5H). Furthermore, the methylated patterns among Upstream-, Exonic-, Intronic-, Downstream- and Intergenic-CGIs were likely to display in a stage-specific manner for the Pre-, In- and Post-pubertal stage in the CpH context (Fig 5E, 5F and 5G).

### Change patterns of DNA methylation among these pituitaries

**Fig 6:**
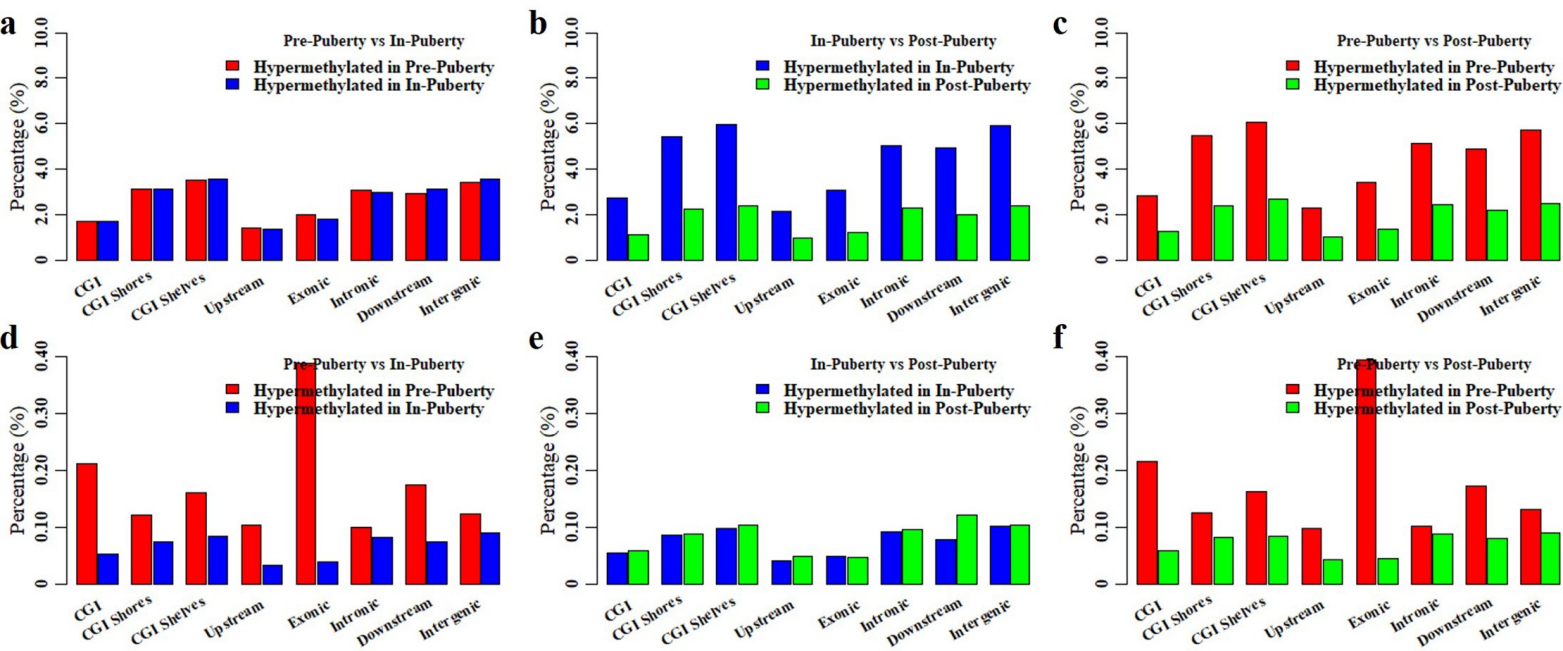
Differentially methylated CpGs and CpHs among these pituitaries. Distributions of differentially methylated CpGs between Pre- and In- (**a**), In- and Post- (**b**), and Pre- and Post-pubertal methylome (**c**). Distributions of differentially methylated CpHs between Pre- vs In- (**d**), In- vs Post- (**e**), and Pre- vs Post-pubertal stage (**f**).

To explore whether the DNA methylation changes among these pituitaries preferentially happen at certain genomic regions, the distributions of the differentially methylated CpGs (DMGs) and CpHs (DMHs) were shown in Fig 6. Respectively, 68237, 80567, and 83225 DMGs were identified for Pre- vs. In- (Fig 6A), In- vs. Post- (Fig 6B) and Pre- vs. Post-puberty (Fig 6C). Pre-pubertal stage appeared to show the same number of hyper-DMGs with that in In-pubertal methylome (Fig 6A). Compared with Post-pubertal stage, more hyper-DMGs were found in Pre- and In-stage (Fig 6A, 6B and 6C). Moreover, DMGs were likely to be underrepresented in CGI, upstream and exonic regions (relative enrichment = 0.46–0.70, *P* < 2.22 × 10^−16^) (Table 1), but overrepresented in CGI shores, CGI shelves, intronic, downstream and intergenic regions (relative enrichment = 1.12–1.51, *P* < 2.22 × 10^−16^) (Table 1).

**Table 1:** Distribution of differentially methylated CpGs among these pituitaries.

	Detected CpGs	DMGs between Pre- and In-puberty		DMGs between In- and Post-puberty		DMGs between Pre- and Post-puberty	
		Number	Enrichment	Number	Enrichment	Number	Enrichment
**Total**	1,301,936	68,237	—	80,567	—	83,225	—
**CGI**	581,907	19,650	0.50 (*P* < 2.22 × 10^−16^)	22,352	0.48 (*P* < 2.22 × 10^−16^)	23,791	0.50 (*P* < 2.22 × 10^−16^)
**CGI shores**	264,273	16,423	1.24 (*P* < 2.22 × 10^−16^)	20,242	1.32 (*P* < 2.22 × 10^−16^)	20,805	1.31 (*P* < 2.22 × 10^−16^)
**CGI shelves**	87,971	6,209	1.38 (*P* < 2.22 × 10^−16^)	7,379	1.39 (*P* < 2.22 × 10^−16^)	7,690	1.40 (*P* < 2.22 × 10^−16^)
**Upstream**	213,133	5,881	0.48 (*P* < 2.22 × 10^−16^)	6,599	0.46 (*P* < 2.22 × 10^−16^)	7,096	0.48 (*P* < 2.22 × 10^−16^)
**Exon**	278,535	10,523	0.67 (*P* < 2.22 × 10^−16^)	11,919	0.64 (*P* < 2.22 × 10^−16^)	13,269	0.70 (*P* < 2.22 × 10^−16^)
**Intron**	420,362	25,453	1.25 (*P* < 2.22 × 10^−16^)	30,674	1.29 (*P* < 2.22 × 10^−16^)	31,726	1.29 (*P* < 2.22 × 10^−16^)
**Downstream**	74,434	4,517	1.17 (*P* < 2.22 × 10^−16^)	5,154	1.13 (*P* < 2.22 × 10^−16^)	5,276	1.12 (*P* < 2.22 × 10^−16^)
**Intergenic**	315,472	21,863	1.47 (*P* < 2.22 × 10^−16^)	26,221	1.51 (*P* < 2.22 × 10^−16^)	25,858	1.41 (*P* < 2.22 × 10^−16^)

Enrichment of differentially methylated CpGs for certain genomic regions was calculated by using with a two-tail Fisher’s exact test.

Furthermore, 12959, 9404, and 13335 DMHs were identified for Pre- vs. In- (Fig 6D), In- vs. Post- (Fig 6E) and Pre- vs. Post-puberty (Fig 6F), respectively. In-pubertal stage appeared to show the same number of hyper-DMHs with that in Post-pubertal stage (Fig 6D). However, compared with Pre-pubertal stage, less hyper-DMHs were found in In- and Post-stage (Fig 6E and 6F). Moreover, the DMHs were likely to show an overrepresentation in CGI shelves and downstream regions (relative enrichment = 1.09–1.22, *P* ≤ 3.06 × 10^−3^) (Table 2) but show an underrepresentation in upstream (relative enrichment = 0.51–0.58, *P* < 2.22 × 10^−16^) (Table 2). Importantly, these DMHs exhibited in a stage-specific enrichment for CGI, CGI shores, exonic, intronic and intergenic regions (Table 2).

**Table 2:** Distribution of differentially methylated CpHs among these pituitaries.

	Detected CpHs	DMHs between Pre- and In-puberty		DMHs between In- and Post-puberty		DMHs between Pre- and Post-puberty	
		Number	Enrichment	Number	Enrichment	Number	Enrichment
**Total**	5,708,233	12,959	—	9,404	—	13,335	—
**CGI**	1,443,684	3,788	1.22 (*P* < 2.22 × 10^−16^)	1,603	0.61 (*P* < 2.22 × 10^−16^)	3,934	1.29 (*P* < 2.22 × 10^−16^)
**CGI shores**	1,488,072	2,882	0.81 (*P* < 2.22 × 10^−16^)	2,579	1.07 (*P* = 2.94 × 10^−3^)	3,064	0.88 (*P* = 2.26 × 10^−10^)
**CGI shelves**	494,636	1,211	1.09 (*P* = 6.53 × 10^−3^)	991	1.13 (*P* = 5.26 × 10^−14^)	1,210	1.09 (*P* = 7.18 × 10^−3^)
**Upstream**	755,007	1,024	0.56 (*P* < 2.22 × 10^−16^)	6,73	0.51 (*P* < 2.22 × 10^−16^)	1,055	0.58 (*P* < 2.22 × 10^−16^)
**Exon**	890,620	3,799	2.24 (*P* < 2.22 × 10^−16^)	846	0.53 (*P* < 2.22 × 10^−16^)	3,900	2.33 (*P* < 2.22 × 10^−16^)
**Intron**	2,084,096	3,785	0.72 (*P* < 2.22 × 10^−16^)	3,853	1.07 (*P* = 1.46 × 10^−3^)	3,928	0.76 (*P* < 2.22 × 10^−16^)
**Downstream**	361,176	893	1.10 (*P* = 9.29 × 10^−3^)	716	1.22 (*P* = 6.24 × 10^−7^)	903	1.11 (*P* = 3.06 × 10^−3^)
**Intergenic**	1,617,334	3,485	0.93 (*P* = 2.62 × 10^−4^)	3,316	1.38 (*P* < 2.22 × 10^−16^)	3,549	0.95 (*P* = 1.68 × 10^−2^)

Enrichment of differentially methylated CpHs for certain genomic regions was calculated by using with a two-tail Fisher’s exact test.

### Biological functions of DNA methylation changes among these pituitaries

**Fig 7:**
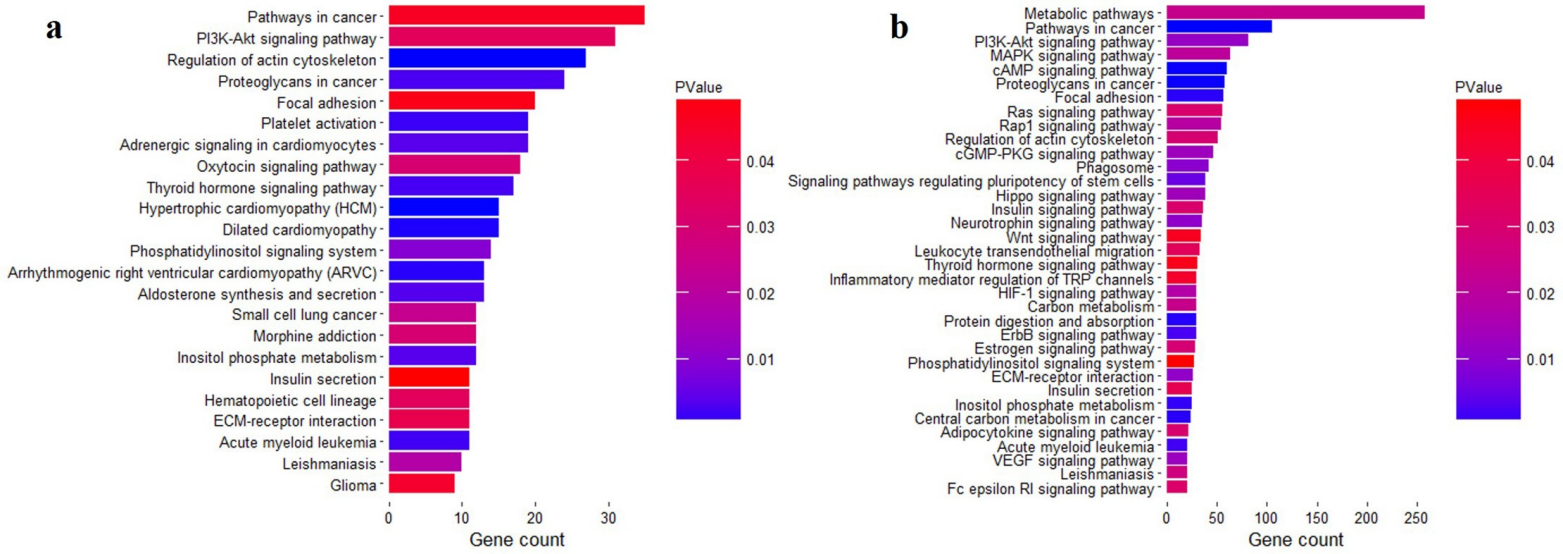
KEGG enrichment analysis of DNA methylation changes. (**a**) The significantly enriched signaling pathway of genes associating with CpG methylation changes. (**b**) The significantly enriched signaling pathway of genes associating with CpH methylation changes.

To gain insight into the biological functions and processes in which genes associated with the DNA methylation changes might be involved, we performed the GO and KEGG enrichment analysis on genes who were associated with DMGs or DMHs (see methods and materials). During pubertal transition, 4301 genes were identified as the DMG genes in the pituitaries (S1 File). The most significant terms of genes associated with DMGs were negative regulation of transcription, regulation of cell proliferation, and cell growth (S1A Fig). Moreover, these genes were also enriched in PI3K-Akt signaling pathway, Oxytocin signaling pathway, and Insulin secretion (Fig 7A). These pathways were highly involved in the fundamental biological function of pituitary tissues. Furthermore, 3691 genes were identified as the DMHs genes in these pituitaries (S2 File). The most significantly enriched terms of genes associated with DMHs were regulation of cell proliferation, cell maturation, development (such as in utero embryonic development, kindey development, skin development and regulation of projection development), and morphogenesis (such as inner ear morphogenesis and embryonic limb morphogenesis) (S1B Fig). These genes were enriched in Metabolic pathways, Insulin signaling pathway, Neurotrophin signaling pathway, and Estrogen signaling pathway (Fig 7B). These pathways were likely to be more highly involved in the releasing gonadotropin hormones from pituitary tissues during the timing of puberty in pigs.

## Discussion

During the timing of puberty, the pituitary has been thought to connect with and respond to the hypothalamus and then introduce the following response of ovary in HPG axis [38, 39]. Previous studies demonstrated that disrupted DNA methylation results in the delayed puberty [22, 24]. The DNA methylation of pituitaries was supposed to show dynamics and changes during the pubertal transition in mammals. In the present study, the genome-wide DNA methylation profiles were profiled for the pituitary tissues of Pre-, In- and Post-pubertal gilts. We found that the average methylation level of CpGs in Post- was lower than that in Pre- and In-puberty (Fig 1B and 1C), but the average methylation level of CpHs in In- appeared to higher than that in Pre- and Post-puberty (Fig 1E and 1F). Besides, compared with that in Pre- and In-stage, the CpG methylation level was the lowest at the gene and CGI locations (Fig 3A and 3B) in Post-stage. However, the methylation levels of CpHs were likely to be overlapped together across Pre-, In- and Post-puberty at gene and CGI locations (Fig 3C and 3D). The similar observations were also found in the location of HCP and LCP genes (Fig 4). These results recommended that changes of CpG methylation were different from that of CpHs among these pituitaries during the timing of puberty.

We found that the Pearson’s correlation coefficients of the global methylation were all 0.99 (*P* < 2.22 × 10^−16^) for the comparisons of Pre- vs. In-, In- vs. Post-, and Pre- vs. Post-pubertal pituitaries in the CpG context (Fig 2A), but the Pearson’s correlation coefficient was 0.57, 0.80 and 0.58 in CpH context, respectively (Fig 2B). Moreover, the Person’s correlation coefficients of the methylation patterns of CpGs were all 0.99 at the locations of genes and CGIs for the comparisons of Pre- vs. In-, Pre- vs. Post- and In- vs. Post-puberty, respectively (Fig 3A and 3B), but the Person’s correlation coefficient of CpH methylation patterns was 0.88, 0.81 and 0.95 at the locations of genes (Fig 3C and S2 Table) and was 0.92, 0.88 and 0.98 at the locations of CGIs, respectively (Fig 3D and S2 Table). Besides, in the CpG context, the Person’s correlation coefficients of the methylation patterns were all 0.99 against Pre- vs. In-, In- vs. Post-, and Pre- vs. Post-puberty at the locations of HCP and LCP genes, respectively. However, the Person’s correlation coefficients between the CpH methylation patterns were 0.85, 0.93 and 0.80 for HCP genes, and 0.86, 0.94 and 0.79 for LCP genes against Pre- vs. In-, In- vs. Post-, and Pre- vs. Post-puberty, respectively (Fig 4C and 4D and S3 Table). These results indicated that the CpH methylation were more dynamic than CpG methylation among these pituitaries during the timing of puberty.

Totally, 12.65% of detected CpGs were identified as the DMGs among these pituitaries. These DMGs appeared to be hypermethylated in Pre- and In-pubertal stages but hypomethylated in Post-pubertal stages (Fig 6A, 6B and 6C). This result was in line with the observation that that the methylation level of Post-pubertal stage was lower than that in Pre- and Post-pubertal stage at the locations of whole genes (Fig 3A), CGIs (Fig 3B), HCP genes (Fig 4A) and LCP genes (Fig 4B). Moreover, the enrichments of these DMGs ranged from 0.46 to 0.70 at CGI, upstream and exonic regions (Table 1), which were much lower than the enrichments of DMGs located at CGI shores, CGI shelves, intronic, downstream and intergenic regions (ranging from 1.12 to 1.51) (Table 1). Alternatively, DMGs among Pre- and In-, In- and Post-, and Pre- and Post-pubertal methylome were likely to show the similarly enriched patterns (Table 1). This result was in accordance with the exhibition that the methylation patterns among Upstream-, Exonic-, Intronic-, Downstream- and Intergenic-CGIs showed in the same manner for the Pre-, In- and Post-pubertal stage (Fig 5A, 5B and 5C). These observations indicated that the CpG methylation changes were likely to show in a similar pattern during the timing of puberty.

Furthermore, 0.35% of detected CpHs were identified as DMHs among these pituitaries. These DMHs appeared to by hypermethylated in Pre-pubertal stage but hypomethylated in In- and Post-pubertal stage (Fig 6D, 6E and 6F). The relative enrichment of DMHs, ranging from 1.09 to 1.22, recommended an overrepresentation in CGI shelves and downstream regions (Table 1), and an underrepresentation in upstream region with the enrichment ranging from 0.51 to 0.58 (Table 2). These results were in line with the finding that observed in DMGs (S3 Table). However, these DMHs exhibited in a stage-specific enrichment for CGI, CGI shores, exonic, intronic and intergenic region (Table 2). Moreover, these stage-specific dynamics were also observed at the methylation patterns among Exonic-, Intronic- and Intergenic-CGI at Pre- (Fig 5E), In- (Fig 5F) and Post-pubertal stage (Fig 5G). These results suggested that the CpH methylation changes were likely to show the stage-specific pattern during the onset and timing of puberty.

The biological processes and pathway of the DNA methylation changes were explored during the timing of puberty. We found that genes associating with CpG methylation changes were enriched the regulation of transcription, regulation of cell proliferation and differentiation, PI3K-Akt signaling pathway, Oxytocin signaling pathway, and Insulin secretion (Fig 7A), which were more likely to be involved in the fundamental functions of pituitary tissues. However, genes associated with CpH methylation changes were enriched in Insulin signaling pathway, Neurotrophin signaling pathway and Estrogen signaling pathway (Fig 7A), which were more likely to get involved in the biological functions of releasing gonadotropin hormones from pituitary tissues during the timing of puberty; that is the pituitary responded to the hypothalamus and then released gonadotropin hormones to introduce the following response of ovary in HPG axis.

## Supporting information

S1 Fig GO term analysis of DNA methylation changes. (**a**) The significantly enriched GO terms of biological processes of genes associating with CpG methylation changes. (**b**) The significantly enriched GO terms of biological processes of genes associating with CpH methylation changes.
(JPG)

S1 Table Correlation coefficients of methylation levels with densities of genes and CGIs. (DOCX)

S2 Table Correlation coefficients of methylated patterns and densities of CpHs at genic locations. (DOCX)

S3 Table Correlation coefficients of methylation patterns and densities of CpHs at the locations of HCP and LCP genes. (DOCX)

S1 File The list of genes regarding to DMGs. (CSV)

S2 File The list of genes regarding to DMHs. (CSV)

## References

[1] Choi JH, Yoo HW. Control of puberty: genetics, endocrinology, and environment. Curr Opin Endocrinol Diabetes Obes. 2013;20(1):62–8. 10.1097/MED.0b013e32835b7ec7 . 23183357

[2] Gajdos ZK, Hirschhorn JN, Palmert MR. What controls the timing of puberty? An update on progress from genetic investigation. Curr Opin Endocrinol Diabetes Obes. 2009;16(1):16–24. 10.1097/MED.0b013e328320253c . 19104234

[3] Zhu J, Chan YM. Adult Consequences of Self-Limited Delayed Puberty. Pediatrics. 2017;139(6). 10.1542/peds.2016-3177 . 28562264 PMC8579478

[4] Howard SR. Genes underlying delayed puberty. Mol Cell Endocrinol. 2018 10.1016/j.mce.2018.05.001 . 29730183 PMC6127442

[5] Bramswig J, Dubbers A. Disorders of pubertal development. Dtsch Arztebl Int. 2009;106(17):295–303; quiz 4. 10.3238/arztebl.2009.0295 19547638 PMC2689583

[6] Coe CL, Chen J, Lowe EL, Davidson JM, Levine S. Hormonal and behavioral changes at puberty in the squirrel monkey. Horm Behav. 1981;15(1):36–53. . 7216188 10.1016/0018-506x(81)90033-7

[7] Root AW. Hormonal changes in puberty. Pediatr Ann. 1980;9(10):365–75. . 6777745

[8] Dutta S, Mark-Kappeler CJ, Hoyer PB, Pepling ME. The steroid hormone environment during primordial follicle formation in perinatal mouse ovaries. Biol Reprod. 2014;91(3):68 10.1095/biolreprod.114.119214 . 25078683

[9] Richards JS. The Ovarian Cycle. Vitam Horm. 2018;107:1–25. 10.1016/bs.vh.2018.01.009 . 29544627

[10] de Jesus-Silva LM, de Oliveira PV, da Silva Ribeiro C, Ninhaus-Silveira A, Verissimo-Silveira R. Ovarian cycle in Devario aequipinnatus with emphasis on oogenesis. Zygote. 2018:1–9. 10.1017/S0967199418000060 . 29607795

[11] Couldrey C, Brauning R, Bracegirdle J, Maclean P, Henderson HV, McEwan JC. Genome-wide DNA methylation patterns and transcription analysis in sheep muscle. PLoS One. 2014;9(7):e101853 10.1371/journal.pone.0101853 25010796 PMC4092064

[12] Hartung T, Zhang L, Kanwar R, Khrebtukova I, Reinhardt M, Wang C, et al Diametrically opposite methylome-transcriptome relationships in high- and low-CpG promoter genes in postmitotic neural rat tissue. Epigenetics-Us. 2012;7(5):421–8. 10.4161/epi.19565 22415013 PMC3368807

[13] Meissner A, Mikkelsen TS, Gu H, Wernig M, Hanna J, Sivachenko A, et al Genome-scale DNA methylation maps of pluripotent and differentiated cells. Nature. 2008;454(7205):766–70. 10.1038/nature07107 18600261 PMC2896277

[14] Mikkelsen TS, Ku M, Jaffe DB, Issac B, Lieberman E, Giannoukos G, et al Genome-wide maps of chromatin state in pluripotent and lineage-committed cells. Nature. 2007;448(7153):553–60. 10.1038/nature06008 17603471 PMC2921165

[15] Illingworth RS, Gruenewald-Schneider U, Webb S, Kerr AR, James KD, Turner DJ, et al Orphan CpG islands identify numerous conserved promoters in the mammalian genome. PLoS Genet. 2010;6(9):e1001134 10.1371/journal.pgen.1001134 20885785 PMC2944787

[16] Bell JSK, Vertino PM. Orphan CpG islands define a novel class of highly active enhancers. Epigenetics-Us. 2017;12(6):449–64. 10.1080/15592294.2017.1297910 28448736 PMC5501197

[17] Yuan XL, Zhang Z, Li B, Gao N, Zhang H, Sangild PT, et al Genome-wide DNA methylation analysis of the porcine hypothalamus-pituitary-ovary axis. Sci Rep. 2017;7(1):4277 10.1038/s41598-017-04603-x 28655931 PMC5487323

[18] Lomniczi A, Wright H, Ojeda SR. Epigenetic regulation of female puberty. Front Neuroendocrinol. 2015;36:90–107. 10.1016/j.yfrne.2014.08.003 25171849 PMC6824271

[19] Ojeda SR, Lomniczi A. Puberty in 2013: Unravelling the mystery of puberty. Nat Rev Endocrinol. 2014;10(2):67–9. 10.1038/nrendo.2013.233 . 24275741

[20] Leka-Emiri S, Chrousos GP, Kanaka-Gantenbein C. The mystery of puberty initiation: genetics and epigenetics of idiopathic central precocious puberty (ICPP). J Endocrinol Invest. 2017;40(8):789–802. 10.1007/s40618-017-0627-9 . 28251550

[21] Adekunbi DA, Li XF, Li S, Adegoke OA, Iranloye BO, Morakinyo AO, et al Role of amygdala kisspeptin in pubertal timing in female rats. PLoS One. 2017;12(8):e0183596 10.1371/journal.pone.0183596 28846730 PMC5573137

[22] Toro CA, Aylwin CF, Lomniczi A. Hypothalamic Epigenetics Driving Female Puberty. J Neuroendocrinol. 2018 10.1111/jne.12589 . 29520866 PMC6043392

[23] Lomniczi A, Ojeda SR. The Emerging Role of Epigenetics in the Regulation of Female Puberty. Endocr Dev. 2016;29:1–16. 10.1159/000438840 26680569 PMC4955615

[24] Lomniczi A, Loche A, Castellano JM, Ronnekleiv OK, Bosch M, Kaidar G, et al Epigenetic control of female puberty. Nat Neurosci. 2013;16(3):281–9. 10.1038/nn.3319 23354331 PMC3581714

[25] Yang C, Ye J, Liu Y, Ding J, Liu H, Gao X, et al Methylation pattern variation between goats and rats during the onset of puberty. Reprod Domest Anim. 2018;53(3):793–800. 10.1111/rda.13172 . 29577480

[26] Yang C, Ye J, Li X, Gao X, Zhang K, Luo L, et al DNA Methylation Patterns in the Hypothalamus of Female Pubertal Goats. PLoS One. 2016;11(10):e0165327 10.1371/journal.pone.0165327 27788248 PMC5082945

[27] Luo L, Yao Z, Ye J, Tian Y, Yang C, Gao X, et al Identification of differential genomic DNA Methylation in the hypothalamus of pubertal rat using reduced representation Bisulfite sequencing. Reprod Biol Endocrinol. 2017;15(1):81 10.1186/s12958-017-0301-2 28985764 PMC5639587

[28] Martinat-Botte F, Royer E, Venturi E, Boisseau C, Guillouet P, Furstoss V, et al Determination by echography of uterine changes around puberty in gilts and evaluation of a diagnosis of puberty. Reprod Nutr Dev. 2003;43(3):225–36. . 14620630 10.1051/rnd:2003022

[29] Soede NM, Langendijk P, Kemp B. Reproductive cycles in pigs. Anim Reprod Sci. 2011;124(3–4):251–8. 10.1016/j.anireprosci.2011.02.025 . 21397415

[30] Djahanbakhch O, Ezzati M, Zosmer A. Reproductive ageing in women. J Pathol. 2007;211(2):219–31. 10.1002/path.2108 . 17200943

[31] Patterson JL, Willis HJ, Kirkwood RN, Foxcroft GR. Impact of boar exposure on puberty attainment and breeding outcomes in gilts. Theriogenology. 2002;57(8):2015–25. . 12066862 10.1016/s0093-691x(02)00674-x

[32] Yuan XL, Zhang Z, Pan RY, Gao N, Deng X, Li B, et al Performances of Different Fragment Sizes for Reduced Representation Bisulfite Sequencing in Pigs. Biol Proced Online. 2017;19:5 10.1186/s12575-017-0054-5 28596713 PMC5463379

[33] Yuan XL, Gao N, Xing Y, Zhang HB, Zhang AL, Liu J, et al Profiling the genome-wide DNA methylation pattern of porcine ovaries using reduced representation bisulfite sequencing. Sci Rep. 2016;6:22138 10.1038/srep22138 26912189 PMC4766444

[34] Krueger F, Andrews SR. Bismark: a flexible aligner and methylation caller for Bisulfite-Seq applications. Bioinformatics. 2011;27(11):1571–2. 10.1093/bioinformatics/btr167 21493656 PMC3102221

[35] Gu H, Bock C, Mikkelsen TS, Jager N, Smith ZD, Tomazou E, et al Genome-scale DNA methylation mapping of clinical samples at single-nucleotide resolution. Nat Methods. 2010;7(2):133–U69. 10.1038/nmeth.1414 WOS:000274086200020. 20062050 PMC2860480

[36] Guo W, Zhu P, Pellegrini M, Zhang MQ, Wang X, Ni Z. CGmapTools improves the precision of heterozygous SNV calls and supports allele-specific methylation detection and visualization in bisulfite-sequencing data. Bioinformatics. 2018;34(3):381–7. 10.1093/bioinformatics/btx595 . 28968643 PMC6454434

[37] Jiao X, Sherman BT, Huang da W, Stephens R, Baseler MW, Lane HC, et al DAVID-WS: a stateful web service to facilitate gene/protein list analysis. Bioinformatics. 2012;28(13):1805–6. 10.1093/bioinformatics/bts251 22543366 PMC3381967

[38] Sellix MT. Clocks underneath: the role of peripheral clocks in the timing of female reproductive physiology. Front Endocrinol (Lausanne). 2013;4:91 10.3389/fendo.2013.00091 23888155 PMC3719037

[39] Abreu AP, Kaiser UB. Pubertal development and regulation. Lancet Diabetes Endocrinol. 2016;4(3):254–64. 10.1016/S2213-8587(15)00418-0 26852256 PMC5192018

