# Supplementary figures and images for: Genome-wide DNA methylation analysis of pituitaries during the initiation of puberty in gilts
Source: PLoS One. 2019 Mar 7;14(3):e0212630. doi: 10.1371/journal.pone.0212630 (PMC6405085; doi:10.1371/journal.pone.0212630)

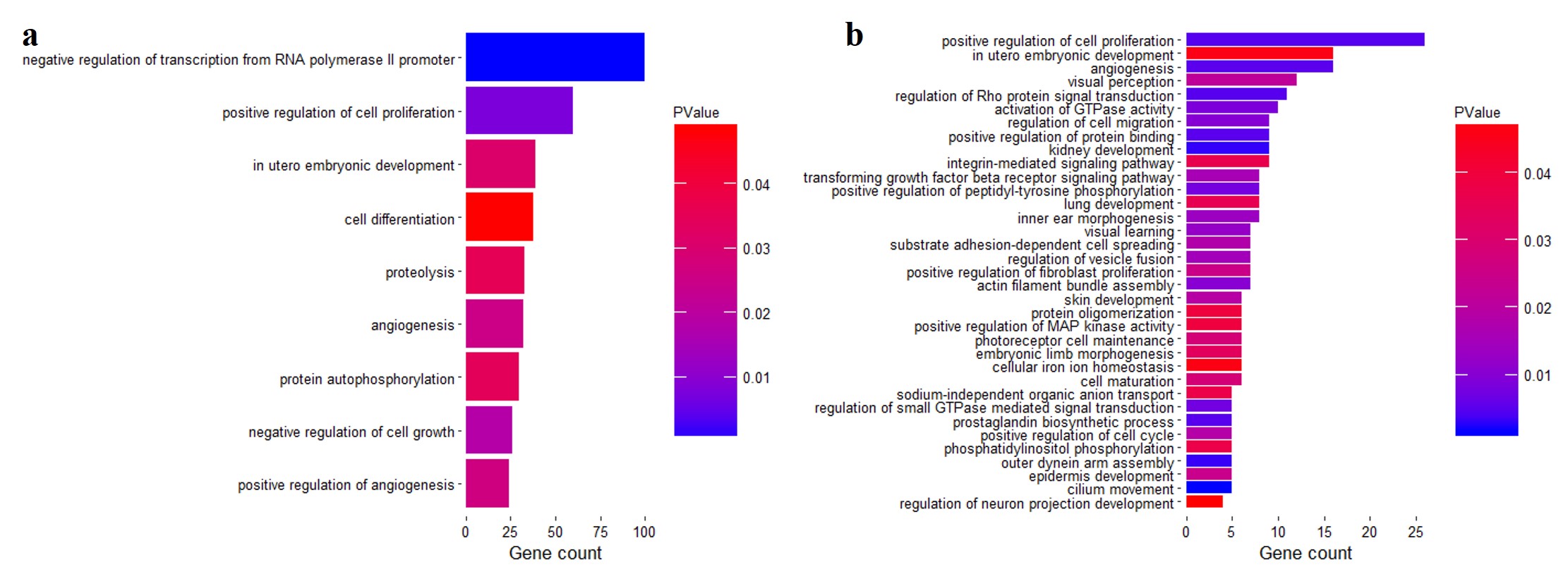

Supplement: S1 Fig — (a) The significantly enriched GO terms of biological processes of genes associating with CpG methylation changes. (b) The significantly enriched GO terms of biological processes of genes associating with CpH methylation changes. (JPG) [file pone.0212630.s001.jpg]
