# Supplementary material for: Genome-wide DNA methylation analysis of pituitaries during the initiation of puberty in gilts
Source: PLoS One. 2019 Mar 7;14(3):e0212630. doi: 10.1371/journal.pone.0212630 (PMC6405085; doi:10.1371/journal.pone.0212630)
Supplement: S1 Table — (DOCX) [file pone.0212630.s002.docx]

**S1 Table. Correlation coefficients of methylation levels with densities of genes and CGIs**

|  | | **DNA methylomes** | | | **Densities of genes** | **Densities of CGIs** | **Densities of CpHs** |
| --- | --- | --- | --- | --- | --- | --- | --- |
|  |  | **Pre-puberty** | **In-puberty** | **Post-puberty** |  |  |  |
| **DNA methylomes** | **Pre-puberty** | — | 0.56 (*P* < 2.22 × 10^−16^) | 0.58 (*P* < 2.22 × 10^−16^) | -0.01 (*P* = 0.96) | 0.07 (*P* = 9.38 × 10^−4^) | 0.09 (*P* = 3.99 × 10^−4^) |
|  | **In-puberty** | 0.99 (*P* < 2.22 × 10^−16^) | — | 0.80 (*P* < 2.22 × 10^−16^) | -0.07 (*P* = 6.28 × 10^−4^) | 0.15 (*P* = 4.95 × 10^−13^) | 0.15 (*P* = 6.63 × 10^−14^) |
|  | **Post-puberty** | 0.99 (*P* < 2.22 × 10^−16^) | 0.99 (*P* < 2.22 × 10^−16^) | — | -0.06 (*P* = 7.03 × 10^−3^) | 0.14 (*P* = 1.20 × 10^−6^) | 0.15 (*P* = 3.03 × 10^−13^) |
| **Densities of genes** | | -0.12 (*P* < 2.22 × 10^−16^) | -0.12 (*P* < 2.22 × 10^−16^) | -0.12 (*P* < 2.22 × 10^−16^) | — | 0.42 (*P* < 2.22 × 10^−16^) | 0.55 (*P* < 2.22 × 10^−16^) |
| **Densities of CGIs** | | 0.27 (*P* < 2.22 × 10^−16^) | 0.27 (*P* < 2.22 × 10^−16^) | 0.27 (*P* < 2.22 × 10^−16^) | 0.42 (*P* < 2.22 × 10^−16^) | — | 0.60 (*P* < 2.22 × 10^−16^) |
| **Densities of CpGs** | | 0.22 (*P* < 2.22 × 10^−16^) | 0.22 (*P* < 2.22 × 10^−16^) | 0.22 (*P* < 2.22 × 10^−16^) | 0.50 (*P* < 2.22 × 10^−16^) | 0.88 (*P* < 2.22 × 10^−16^) | — |

Correlation coefficients were calculated by Pearson’s correlation.

The lower triangle represents the correlation coefficients of CpG methylation, and the upper triangle represents the correlation coefficients of CpH methylation.

CGIs: CpG islands.
