# Supplementary material for: Genome-wide DNA methylation analysis of pituitaries during the initiation of puberty in gilts
Source: PLoS One. 2019 Mar 7;14(3):e0212630. doi: 10.1371/journal.pone.0212630 (PMC6405085; doi:10.1371/journal.pone.0212630)
Supplement: S2 Table — (DOCX) [file pone.0212630.s003.docx]

**S2 Table. Correlation coefficients of methylation patterns and densities of CpHs at genic locations**

|  | **Pre-puberty** | **In-puberty** | **Post-puberty** | **Densities of CpHs** |
| --- | --- | --- | --- | --- |
| **Pre-puberty** | — | 0.92 (*P* < 2.22 × 10^−16^) | 0.89 (*P* < 2.22 × 10^−16^) | -0.59 (*P* = 7.56 × 10^−7^) |
| **In-puberty** | 0.88 (*P* < 2.22 × 10^−16^) | — | 0.97 (*P* < 2.22 × 10^−16^) | -0.78 (*P* = 2.16 × 10^−13^) |
| **Post-puberty** | 0.81 (*P* < 2.22 × 10^−16^) | 0.95 (*P* < 2.22 × 10^−16^) | — | -0.80 (*P* = 7.54 × 10^−15^) |
| **Densities of CpHs** | -0.17 (*P* = 0.14) | -0.20 (*P* = 0.07) | -0.28 (*P* = 0.01) | — |

Correlation coefficients were calculated by Pearson’s correlation.

The lower triangle represents the correlation coefficient of CpH methylation at genic locations, and the upper triangle represents the correlation coefficient of CpH methylation at CGI locations. CGIs: CpG islands.
