# Supplementary material for: Genome-wide DNA methylation analysis of pituitaries during the initiation of puberty in gilts
Source: PLoS One. 2019 Mar 7;14(3):e0212630. doi: 10.1371/journal.pone.0212630 (PMC6405085; doi:10.1371/journal.pone.0212630)
Supplement: S3 Table — (DOCX) [file pone.0212630.s004.docx]

**S3 Table. Correlation coefficients of methylation patterns and densities of CpHs at the locations of HCP and LCP genes**

|  | **Pre-puberty** | **In-puberty** | **Post-puberty** | **Densities of CpHs** |
| --- | --- | --- | --- | --- |
| **Pre-puberty** | — | 0.86 (*P* < 2.22 × 10^−16^) | 0.79 (*P* < 2.22 × 10^−16^) | -0.36 (*P* = 1.14 × 10^−3^) |
| **In-puberty** | 0.85 (P < 2.22 × 10^−16^) | — | 0.94 (P < 2.22 × 10^−16^) | -0.43 (*P* = 7.93 × 10^−5^) |
| **Post-puberty** | 0.80 (P < 2.22 × 10^−16^) | 0.93 (P < 2.22 × 10^−16^) | — | -0.51 (*P* = 1.59 × 10^−6^) |
| **Densities of CpHs** | 0.13 (P = 0.25) | 0.16 (P = 0.20) | 0.13 (P = 0.26) | — |

Correlation coefficients were calculated by Pearson’s correlation.

The lower triangle represents the correlation coefficient of CpH methylation at locations of HCP genes, and the upper triangle represents the correlation coefficient of CpH methylation at locations of LCP genes. HCP: high CpG content promoter; LCP: low CpG content promoter.
